# The relationship between visual hallucinations, functioning and suicidality over the course of illness: a 10-year follow-up study in first-episode psychosis

**DOI:** 10.1192/j.eurpsy.2023.949

**Published:** 2023-07-19

**Authors:** I. Kreis, K. Fjelnseth Wold, G. Åsbø, C. Simonsen, I. Melle

**Affiliations:** 1 NORMENT, Institute of Clinical Medicine, University of Oslo; 2 NORMENT, Division of Mental Health and Addiction, Oslo University Hospital, Oslo, Norway

## Abstract

**Introduction:**

Visual hallucinations are a common symptom across psychotic disorders and have been linked to illness severity, impaired functioning, and increased suicide risk. However, little is known about the stability of this relationship over the long-term course of illness.

**Objectives:**

This study aims to assess whether the presence of visual hallucinations is associated with illness severity, functioning and suicidality, early and late in the course of illness. It further explores the potential role of childhood trauma in this context, which has been linked to both visual hallucinations and suicidality.

**Methods:**

A sample of 185 individuals with first-episode psychosis was assessed with structured clinical interviews and self-report questionnaires at time of study inclusion and at 10-year follow-up. Those with lifetime experience of visual hallucinations at inclusion (VH+/+) as well as those where visual hallucinations first developed during the follow-up period (VH-/+) were compared to a group without such experiences (VH-/-). To this end, multinomial logistic regression models were applied, with a range of clinical and demographic variables as predictors.

**Results:**

At time of inclusion, the VH+/+ group had significantly higher symptom severity scores, lower functioning scores, and were more likely to have a history of multiple suicide attempts. There were no such differences between the VH-/+ and the VH-/- group. At follow-up, this pattern of findings partially reversed. Here, only the VH-/+ group differed from the VH-/- group in terms of higher symptom severity scores and lower functioning scores. However, the VH+/+ group was still more likely to report multiple suicide attempts during the follow-up period, whereas VH-/+ did not differ from VH-/-. Notably, childhood trauma scores did not differ between groups.

**Conclusions:**

In line with previous studies, these findings point to an association between visual hallucinations and illness severity, functioning and suicidality. However, this association seems to change over the course of illness. Together, this highlights the relevance of assessing visual hallucinations in the clinical setting and monitoring their development over time.

**Disclosure of Interest:**

None Declared

